# Person- and Situation-Specific Factors in Discounting Science via Scientific Impotence Excuses

**DOI:** 10.5964/ejop.3735

**Published:** 2021-11-30

**Authors:** Tom Rosman, Martin Kerwer, Anita Chasiotis, Oliver Wedderhoff

**Affiliations:** 1 Leibniz Institute for Psychology (ZPID), Trier, Germany; Kingston University, Kingston upon Thames, United Kingdom

**Keywords:** scientific impotence excuse, prior beliefs, epistemic beliefs, conflicting evidence, discounting science, trust in science

## Abstract

Munro (2010, https://doi.org/10.1111/j.1559-1816.2010.00588.x) found that individuals, when confronted with belief-disconfirming scientific evidence, resist this information by concluding that the topic at hand is not amenable to scientific investigation—a scientific impotence excuse. We strived to replicate this finding and to extend this work by analyzing other factors that might lead to scientific impotence excuses. As a person-specific factor, we analyzed the role of epistemic beliefs, and as a situational factor, we focused on the contradictoriness of the evidence at hand. Three sets of hypotheses were preregistered. In an experimental 2 × 3 online study drawing on a general population sample of N = 901 participants, we first assessed our participants’ prior beliefs on the effects of acupuncture versus massaging (pro acupuncture vs. no opinion). One experimental group then read fictitious empirical evidence claiming superiority of acupuncture, another group read evidence speaking against acupuncture, and a third group read conflicting evidence (i.e., a mix of pro- and contra-findings). Scientific impotence excuses were measured by a newly developed questionnaire. Our first hypothesis, which suggested that participants believing in the superiority of acupuncture would make stronger scientific impotence excuses when confronted with belief-disconfirming findings, was confirmed. A second hypothesis suggested that scientific impotence excuses would be stronger when individuals were confronted with evidence exhibiting a “nature” that contradicts their topic-specific epistemic beliefs. This hypothesis was partially supported. A third hypothesis suggested that individuals confronted with conflicting evidence would make stronger scientific impotence excuses, and this was again confirmed. Implications for theory and practice are discussed.

“In the current political climate […] the credibility of scientific evidence is questioned and science is threatened by defunding” (Wingen et al., 2020, p. 8). With these words, Wingen and colleagues (2020) refer to the issue of right-wing populists discounting science via crude, simple, and yet persuasive messages. While focusing on a multitude of topics (climate change probably being the most prominent example), such messages often have one key element in common: Science is portrayed as unable to investigate the issue at hand, which is why the corresponding scientific findings are deemed meaningless. Interestingly, this relates to a psychological concept from 2010, the so-called Scientific Impotence Excuse (SIE; Munro, 2010) hypothesis, which suggests “that people resist belief disconfirming scientific evidence by concluding that the topic of study is not amenable to scientific investigation” (p. 579). Munro (2010) demonstrated that SIEs can be evoked in experimental settings: If individuals were confronted with scientific evidence that disconfirmed their existing beliefs about a homosexuality stereotype (i.e., on whether homosexuality is associated with mental illness; Munro, 2010), they were more likely to indicate that this topic cannot be studied scientifically (compared to participants receiving belief-confirming evidence; Munro, 2010).

Recently, a majority of Munro’s (2010) predictions were conceptually replicated with regard to a topic from educational research (the effects of grade retention on academic achievement; Thomm et al., 2018; Thomm et al., 2019). However, while the incongruity between scientific claims on a specific topic and individual beliefs on that same topic may indeed be a central factor in discounting science, research has, up to now, neglected other factors that might lead to SIEs. Therefore, the present work aims to extend Munro’s (2010) SIE hypothesis by investigating factors other than belief-disconfirming evidence that may contribute to the devaluation of science. As a situational factor, it will focus on the contradictoriness of the evidence presented, and, as person-specific factor, it will investigate the incongruity between individual epistemic beliefs (i.e., individual beliefs about the nature of knowledge; Hofer & Pintrich, 1997; Stahl & Bromme, 2007) and the (epistemic) nature of the scientific evidence at hand.

## Background and Hypotheses

In the following, we first describe the theoretical background of the SIE effect. Subsequently, we develop three sets of pre-registered hypotheses aiming at 1) conceptually replicating Munro’s (2010) predictions in the context of a health-related topic, 2) extending it with regard to epistemic beliefs as a person-specific factor, and 3) broadening its scope by also including a situation-specific factor, the amount of contradictoriness of the evidence presented.

### The Scientific Impotence Excuse According to Munro (2010)

According to Munro (2010), Festinger’s theory of cognitive dissonance (Festinger, 1957) is at the heart of the SIE proposition. Cognitive dissonance theory suggests that feelings of psychological discomfort (i.e., cognitive dissonance) arise when individuals are subject to two or more contradictory cognitions (Festinger, 1957). Imagine John, who strongly believes in the power of acupuncture. John will likely experience cognitive dissonance when discovering a study report which suggests that acupuncture has no positive effects. Since no one wants to remain in a state of psychological discomfort for long, he will strive to quickly overcome this cognitive dissonance. Research has shown that individuals refer to a multitude of means to do so. Changing prior beliefs is but one (for an overview, see Chinn & Brewer, 1993). Other means, which are sometimes termed as “resistance processes” (Munro, 2010), target the devaluation and denigration of the evidence at hand. Hence, with regard to our example above, John may decide to search for flaws in the acupuncture study. We however concede that this approach is time-consuming and probably contradicts John’s motive of *quickly* overcoming cognitive dissonance. Furthermore, Munro (2010) suggests that “in the real world […] people are rarely provided details regarding the methodology of the research” (p. 581), and we would add that many people lack the skills (e.g., research literacy) to evaluate an empirical study’s quality. Munro (2010) therefore further argues that individuals will likely refer to other resistance processes when confronted with belief-contradicting scientific evidence, and suggests that the strategy of “coming to believe that scientific methods are impotent to address the topic of study” (p. 582) might be particularly promising in this regard—the scientific impotence hypothesis^1^1It should be noted that Munro (2010) further differentiates between the scientific impotence *discounting* hypothesis and the scientific impotence *generalization* hypothesis. According to the latter, scientific impotence excuses regarding one topic would generalize to other topics or to the scientific method as a whole (an expectation that was however not empirically supported in Thomm and colleagues’ [2019] study). For reasons of parsimony, the present article only focuses on the scientific impotence discounting hypothesis and ignores this generalization aspect. is born.

As outlined above, a number of studies have confirmed the basic assumption of Munro’s (2010) SIE hypothesis, but, to date, only a limited number of topics have been investigated. In fact, Munro (2010) only tested his predictions with regard to beliefs about a homosexuality stereotype, and Thomm and colleagues’ (2018, 2019) study aimed at the topic of grade retention. To our knowledge, no corresponding studies have been conducted in the health domain, which is striking since SIEs regarding medical knowledge might have very disastrous consequences (imagine, for example, the consequences of SIE in the Corona pandemic). We expect that SIEs are particularly widespread concerning topics from complementary and alternative medicine. In fact, people who refer to alternative medicine are likely to adapt a more “esoteric” and less scientific way of thinking—believing, for example, in paranormal phenomena and in magical effects of food on health (Furnham, 2007; Saher & Lindeman, 2005). Furthermore, proponents and advocates of alternative medicine often argue that their drugs or treatment approaches are not scientifically investigable (e.g., Oberbaum et al., 2003; Power & Hopayian, 2011). They state, for example, that the effect mechanisms are too complex to analyze, or that a scientific investigation would obstruct the proposed healing mechanisms as it “chase[s] the healing process away” (Baum & Ernst, 2009, p. 973). In the present paper, we therefore test the scientific impotence hypothesis in the context of a topic from alternative medicine (acupuncture vs. massaging in the treatment of back pain). Choosing this topic also has the advantage that it currently is not a hot topic in Germany, meaning that most study participants do not have much prior topic knowledge—which is important for the success of corresponding experimental manipulations. In fact, investigating the SIE almost always implies delivering some kind of unbalanced evidence (e.g., only studies speaking in favor of acupuncture). However, if participants doubt that this evidence correctly represents the available evidence because of high prior knowledge, they may rather become suspicious of the experiment instead of referring to SIEs. For these reasons, we chose to investigate SIE regarding the topic of acupuncture versus massaging in the treatment of back pain, and suggest the following confirmatory hypothesis:

*Hypothesis 1:* SIEs will be stronger when individuals are confronted with scientific evidence that contradicts their prior topic-specific beliefs—compared to belief-consistent evidence.

### Scientific Impotence Excuses and Individual Epistemic Beliefs

Epistemic beliefs are individual beliefs about the nature of knowledge and knowing (Hofer & Pintrich, 1997; Stahl & Bromme, 2007), and are often operationalized with regard to a specific academic domain (e.g., psychology-specific epistemic beliefs; Rosman et al., 2017) or topic (e.g., epistemic beliefs on the big-fish-little-pond effect; Merk et al., 2018). Researchers from this field address a multitude of questions relating to individual epistemic beliefs: How do individuals think about knowledge stemming from (e.g., psychological) research? Do they think that it is possible to find out the “truth” about (psychological) phenomena or do they interpret (psychological) “knowledge” as an accumulation of interpretations and opinions? Do they perceive scientific knowledge on the worked-example effect as objective and fixed, or do they see it as tentative and preliminary?^2^2Of note is that there is a certain amount of conceptual overlap between the SIE construct and topic-specific epistemic beliefs. A key difference between both constructs, however, is that SIE arise *in reaction* to a specific stimulus (e.g., scientific evidence that disconfirms one’s existing beliefs), whereas this is not the case with regard to epistemic beliefs. Furthermore, SIE focuses on the potential of scientific methods in generating knowledge, whereas epistemic beliefs more strongly relate to the nature of scientific knowledge itself.


In earlier publications (cf. Buehl et al., 2002; Hofer & Pintrich, 1997; Schommer, 1993; Stahl & Bromme, 2007), there was a more or less general consensus that beliefs about knowledge as certain and fixed (absolute epistemic beliefs) are not very beneficial for learning and information processing. Such publications therefore often suggested that educators should strive to promote views of scientific knowledge as tentative and relative. However, this assumption is not unchallenged. For example, Elby and Hammer (2001) as well as Elby et al. (2016) argue that it strongly depends on the issue in question whether a certain belief may be seen as “correct” (i.e., according to an expert consensus) and “productive” (i.e., beneficial for learning). In line with such arguments, Muis and colleagues (e.g., Muis & Franco, 2010; Muis et al., 2011) suggested what they termed the *consistency hypothesis*: They argued that a congruence between an individual’s epistemic beliefs and the epistemic nature of learning materials fosters metacognitive self-regulation and learning—hence an individual believing in knowledge as tentative and preliminary would learn better with contradictory learning materials (e.g., a text containing conflicting opinions or evidence) than an individual viewing knowledge as an accumulation of absolute “truths.”

Transferring the general idea of the consistency hypothesis (Muis & Franco, 2010; Muis et al., 2011) to the SIE hypothesis, we suggest that inconsistencies between epistemic beliefs and the nature of learning materials may not only lead to poorer self-regulation and learning, but also to dysfunctional attitudes towards science—SIEs. In this context, two extreme cases are possible: Individuals who believe that knowledge is fixed and certain will, when confronted with conflicting evidence, more strongly refer to SIEs compared to individuals who believe that knowledge is tentative and preliminary. Moreover, individuals who believe that knowledge is tentative and preliminary will, if confronted with *non*-conflicting evidence, report more SIEs compared to individuals who believe that knowledge is fixed and certain. Since epistemic beliefs (e.g., beliefs on the nature of knowledge in acupuncture research) and topic-specific beliefs (e.g., beliefs on the efficacy of acupuncture) are conceptually distinct (Kardash & Howell, 2000; Kienhues et al., 2008), we expect that their effects on SIEs are also, at least to a certain extent, independent. In sum, we therefore suggest that inconsistencies between epistemic beliefs and the nature of presented scientific evidence will lead to SIEs—and further expect that these effects are incremental over the “traditional” SIE effects specified by Munro (2010):

*Hypothesis 2:* SIEs will be stronger when individuals are confronted with scientific evidence that exhibits a “nature” contradicting their prior topic-specific epistemic beliefs—compared to belief-consistent evidence (H2a). These effects persist when controlling for “traditional” SIE effects (i.e., the effects of prior topic-specific beliefs; H2b).

### Scientific Impotence Excuses and the Epistemic Nature of Evidence

So far, we have focused on individual factors (e.g., topic-specific beliefs and epistemic beliefs) and on their interaction with situational factors in SIEs. To our knowledge, the effects of situational factors alone, however, have not yet been investigated in the SIE context. This is striking since SIEs exhibit a strong epistemological component. In fact, SIEs can be seen as a lack of beliefs in the epistemology of a domain (Munro, 2010), and, vice-versa, the epistemology of the domain (or topic) itself may also affect SIEs. This is in line with a recent argument by Rosman et al. (2020). In their study, they suggest that students’ epistemic beliefs reflect, to a certain extent, the epistemology of the domain in question, which is why domain-specific epistemic beliefs are usually more absolute with regard to “hard” compared to “soft” (Biglan, 1973) sciences (Hofer, 2000; Muis et al., 2006; Muis et al., 2016; Rosman et al., 2017). SIEs should thus vary depending on the nature of evidence regarding a specific domain or topic.

Furthermore, research has shown that confronting individuals with conflicting scientific evidence (often labelled as “diverging information”) leads to views of scientific evidence as tentative and preliminary (Ferguson et al., 2013; Kienhues et al., 2008; Kienhues et al., 2016). For example, Ferguson et al. (2013) found that their participants tended to express more views of a scientific issue as tentative and complex after being confronted with conflicting information on this same topic (the effects of sun exposure and health, in this case). Such belief changes may be related to SIEs since views of science as strongly tentative imply that any findings are subject to a large level of uncertainty—scientific impotence. In line with these two arguments, we expect that the strength of SIEs depends, to a large extent, on the contradictoriness of the evidence at hand. We further expect that this effect is independent of individual topic-specific beliefs, that only come into play when the evidence supports or contradicts them.

*Hypothesis 3:* SIEs will be stronger when individuals are confronted with contradictory scientific evidence—compared to non-contradictory evidence^3^3It should be noted that Hypotheses 2 and 3 seem to offer opposing predictions at first sight: According to Hypothesis 2, conflicting evidence will lead to less SIE in individuals who believe in knowledge as tentative, whereas Hypothesis 3 predicts that conflicting evidence will lead to more SIE in all participants. However, it may well be that conflicting evidence generally leads to higher SIE compared to non-conflicting evidence (significant main effect of the experimental factor), whereas this difference is somewhat lower in individuals with high relativist beliefs (significant interaction between the experimental factor and the CAEB variable). (H3a). These effects persist when controlling for “traditional” SIE effects (i.e., the effects of prior topic-specific beliefs; H3b).

## Method

Hypotheses were tested in a preregistered experimental study drawing on a German general population sample. The preregistration, which includes all study materials and which was registered on October 15, 2019, can be found in Supplementary Materials.

### Procedure, Design and Materials

The study employed an experimental 3 × 2 between-person design (type of evidence presented × prior beliefs) and was realized in an online format using the survey software EFS Survey^TM^ (“Unipark”). At the very beginning of the questionnaire, participants’ prior topic-specific beliefs (i.e., beliefs on the effectiveness of acupuncture vs. massaging) were assessed using a screening question in a forced-choice format (see preregistration in Supplementary Materials). This allowed us to specify explicit quotas in the survey software, which ensured that we would not run into issues such as having too many participants with no clear opinion on the topic at hand (who can obviously not be confronted with evidence contradicting their prior beliefs). Based on this screening, two equally-sized quasiexperimental groups were formed: 1) Participants believing in the superiority of acupuncture over massaging (“pro acupuncture beliefs” group; QEG1), and 2) participants having no clear opinion on the superiority or inferiority of acupuncture (“no opinion” group; QEG2). To control for gender differences in the perception of acupuncture/massaging, we further specified the quotas so that the gender distribution was identical across the “pro acupuncture” and “no opinion” groups. This is because studies have consistently shown that women more frequently refer to complementary and alternative medicine (e.g., Kristoffersen et al., 2014; Rhee & Harris, 2017; Zhang et al., 2015). Furthermore, participants believing in the superiority of massaging were screened out at the beginning of the data collection since this additional quasiexperimental group was not necessary to test our hypotheses.

After collecting demographics and questionnaire data (e.g., on epistemic beliefs; see Measures section below), we presented our participants with 12 fictitious texts on the treatment of back pain. Eight of these texts described empirical studies on the efficacy of acupuncture versus massaging in back pain treatment, and four additional filler texts described studies irrelevant for the comparison between acupuncture and massaging. All 12 texts were presented on separate pages in the online questionnaire, and their order was randomized. The subset of texts that was presented varied depending on three experimental conditions that the participants were randomly assigned to:

- EG1 (“pro acupuncture”). Participants were presented with four filler texts and eight texts suggesting that acupuncture is better suited for the treatment of back pain than massaging
- EG2 (“against acupuncture”). Participants were presented with four filler texts and eight texts suggesting that massaging is better suited for the treatment of back pain than acupuncture.
- EG3 (“conflicting evidence”). Participants were presented with four filler texts, four “pro acupuncture,” and four “against acupuncture” texts.

The experimental manipulation of the texts was realized by interchanging the words “acupuncture” and “massaging.” This means, for example, that the studies described in EG1 (“pro acupuncture”) and EG2 (“against acupuncture”) were identical except for the fact that the texts in EG2 suggested that massaging was better than acupuncture. The four filler texts were identical across all three conditions.

Text length was around 100 words for each of the 12 texts and all texts were presented in German language (see preregistration in Supplementary Materials). After each text, participants responded to a short comprehension question, in which they were asked to indicate, using a forced-choice format, whether the study suggested that 1) acupuncture is better suited for the treatment of back pain than massaging, 2) massaging is better suited than acupuncture, or that 3) the study is irrelevant for the comparison between acupuncture and massaging (see preregistration in Supplementary Materials).

After this reading task, participants responded to the manipulation checks (see Measures section below) and to a newly developed questionnaire on SIEs (see below), followed by some additional covariates not relevant for the current article (see below). Finally, a debriefing took place. Data collection took around 25 minutes per participant.

### Participants

#### Data Collection Procedures

Participants were recruited using two commercial panel service providers (CINT^TM^ and Respondi^TM^). Participants completed the data collection using their own device, and were paid for their participation by the respective panel provider. Prior to data collection, we pre-specified the following sample properties:

- German speaking participants aged 18–65
- Gender distribution: 50% male, 50% female
- Education: at least middle maturity (“mittlere Reife”^4^4This means that participants needed to have successfully completed at least 10 years of school education. We specified this criterion to ensure that all participants would possess sufficient reading skills to participate in the study.)
- No acupuncture treatments in the last 10 years
- No prior beliefs “against acupuncture” (only “pro acupuncture” and “no opinion”; see Procedure, Design and Materials section)
- Quotas: Gender distribution (50/50, see above) identical across the “pro acupuncture” and “no opinion” groups (see Procedure, Design and Materials section)

#### Sample Size Calculation

Sample size calculation was performed using GPower (Version 3.1; Faul et al., 2009). Since the effects regarding Hypothesis 2 might be smaller than “traditional” SIE effects, especially in an online setting, sample size calculation was performed with a rather small expected effect size of *f* = 0.10. This resulted in a total required sample size of *N* = 967 (*f* = 0.10, α = .05, 1-β = .80, 6 conditions). To reach an optimal distribution of participants across conditions, we aimed to recruit *N* = 972 participants (the next largest number divisible by 4 and 6).

#### Data Screening and Cleaning

In total, *N* = 985 participants completed the online questionnaire. As specified in the preregistration, the raw data were screened, prior to hypothesis testing, for major protocol deviations such as selecting the same response category implausibly often. In a first step, we analyzed our participants’ responses to the 12 comprehension questions on the texts. This revealed rather encouraging results, with 85.8% of participants having at least 10 correct answers, and 70.5% of participants correctly responding to all 12 comprehension questions. Nevertheless, a descriptive glance at the raw data revealed some highly suspicious response patterns in a few participants (such as choosing the exactly same response category across all texts). To address such protocol deviations, we employed the following criterion: Participants were only included in the data analysis if they had responded correctly to at least half of the (fairly easy) comprehension questions on the eight acupuncture-related texts *and* if they had correctly identified at least one distractor. Using this criterion, *n* = 84 participants were excluded from the analyses. This resulted in a final sample size of *N* = 901 participants, with 50.7% women, 49.3% men, and a mean age of *M* = 42.47 (*SD* = 13.52). As specified in our preregistration, we used *z*-scores to screen these data for outliers (criterion: *p*(*z*) < .001), and found no outliers on any (metric) variable relevant for our hypotheses. The actual distribution of participants across experimental and quasiexperimental groups can be found in Table 1. The slight deviation from the pre-specified criterion of *n* = 162 participants per cell was due to simultaneous participation, chance, and, for *n*_cleaned_, the data cleaning procedure.

**Table 1:** Distribution of Participants Among Experimental and Quasiexperimental Groups

Quasiexperimental factor	Experimental factor		
	EG1 (“pro acupuncture” texts)	EG2 (“against acupuncture” texts)	EG3 (texts “conflicting evidence”)
QEG1 (prior beliefs “pro acupuncture”)	*n*_raw_ = 167	*n*_raw_ = 159	*n*_raw_ = 165
	*n*_cleaned_ = 153	*n*_cleaned_ = 147	*n*_cleaned_ = 151
QEG2 (prior beliefs “no opinion”)	*n*_raw_ = 160	*n*_raw_ = 167	*n*_raw_ = 167
	*n*_cleaned_ = 145	*n*_cleaned_ = 150	*n*_cleaned_ = 155

*Note. n*_raw_ = original sample size per group; *n*_cleaned_ = sample size per group after cleaning.

### Measures

#### Scientific Impotence Excuses

SIEs, our main dependent variable, were measured using six newly developed items (see Table 2). To make the instrument as specific as possible, it starts with a short introduction relating all items to the question on whether acupuncture would be better suited for the treatment of back pain than massages. When developing the six items, we focused on covering a broad spectrum of aspects of SIEs, such as problems with the scientific method itself (SIE_01, SIE_04), the general belief that science will not be able to find answers anytime soon (SIE_02), the idea that knowledge has to be constructed by personal experience (SIE_03, SIE_05), or the intricacy of the research questions (SIE_06). As a response format, we opted for a 6-point Likert scale response format as this forces participants to choose whether they agree with the presented SIE statements or not (compared to, for example, a seven-point scale with a middle category). This is because, conceptually, one may either make SIEs or not, and since most of our hypotheses made clear predictions on whether a specific group refers to SIE or not. As expected, an exploratory factor analysis yielded a clear one-dimensional solution (both on scree plots and on the Kaiser-Guttman eigenvalue criterion). Scale reliability (Cronbach’s alpha: α = .89) and item-total correlations (*r*_it_ between .591 and .767) were very good. As specified in our preregistration, scores on individual items were aggregated to mean scores. Higher scores indicate stronger SIE.

**Table 2:** Items and Corrected Item-Total Correlations of the SIE Scale

Label	Item	*r*_it_
(Introduction) Whether acupuncture is better for the treatment of back pain than massages …		
SIE_01	… cannot be investigated by scientific methods.	.748
SIE_02	… will remain hidden from science in the future.	.735
SIE_03	… everyone has to find out for themselves—science cannot provide answers.	.667
SIE_04	… is not amenable to scientific analysis.	.724
SIE_05	… can only be judged by practicing doctors through their experience—not by science.	.591
SIE_06	… depends on so many different influence factors that science cannot find an answer.	.767

*Note.* Response format: 6-point Likert scale ranging from 1 (*do not agree at all*) to 6 (*fully agree*). All items were translated from German language by the authors of the present paper. The English items are not empirically validated. *r*_it_ = corrected item-total correlations for the German items.

#### Epistemic Beliefs

Epistemic beliefs were measured by a slightly adapted version of the Connotative Aspects of Epistemological Beliefs questionnaire (CAEB; Stahl & Bromme, 2007), a well-known inventory that has been used, among others, in studies on the development of epistemic beliefs (e.g., Kienhues et al., 2008; Rosman et al., 2017) as well as in research on the effects of epistemic beliefs on learning (e.g., Bromme et al., 2010). The questionnaire uses 17 adjective pairs and a five-point semantic differential to assess epistemic beliefs on two subscales: texture and variability. For both subscales, higher scores indicate views on knowledge as more tentative and evolving—variability emphasizes the temporal dimension, whereas texture focuses more on the structure of knowledge. To measure epistemic beliefs on a topic-specific level (“acupuncture-specific” epistemic beliefs, so to speak), the instruction of the questionnaire was slightly adapted. After data collection was finished, we conducted an exploratory factor analysis. Contrary to our expectations, scree plot investigations thereby indicated a one-dimensional (instead of two-dimensional) structure. Moreover, reliability analyses yielded unacceptable results regarding the variability subscale (α = .38, *r*_it_ between −.003 and .309), whereas the texture scale seemed to have worked as expected (α = .86). The item-total correlations of this scale were fine, too (*r*_it_ between .486 and .759), except for one item (“ausgehandelt”—“entdeckt”: *r*_it_ = −.038). Consequently, we removed the variability scale from all analyses because of its unacceptable reliability, and proceeded with the original 10-item texture scale (of which a mean score was calculated).

#### Manipulation Checks

Two scales were used to test whether our experimental manipulation had worked as expected. First, a semantic differential single item was administered to assess which general “message” the administered texts had conveyed (7-point Likert-scale with the end points “pro massaging” and “pro acupuncture”; see preregistration in Supplementary Materials). As specified in our preregistration, we expected EG1 (“pro acupuncture” texts) to score higher on this scale compared to EG2 (“against acupuncture” texts) and EG3 (i.e., that they correctly identified their texts as more “pro acupuncture”). Moreover, we expected EG2 to score lower than EG1 (“pro acupuncture”) and EG3 (i.e., that they correctly identified their texts as more “pro massaging / against acupuncture”). Finally, we also expected EG3 scores to be lower than EG1 scores (“pro acupuncture”) but higher than EG2 scores (i.e., that they correctly identified “pro acupuncture” and “pro massaging” texts as more balanced out). Descriptively, these expectations were fully supported (EG1: *M* = 6.55, *SD* = 1.06; EG2: *M* = 1.36, *SD* = 0.70; EG3: *M* = 4.03, *SD* = 1.42), and the corresponding one-factorial analysis of variance confirmed the expected pattern, *F*(2, 898) = 1645.20, *p* < .001, $\eta_{p}^{2}$ = .79, all Tukey HSD post-hoc tests *p* < .001.

Furthermore, we had expected that participants in EG3 (“conflicting evidence”) perceived the presented findings as more inconsistent than the participants in EG1 (“pro acupuncture” texts) and EG2 (“against acupuncture” texts). To test this, a slightly adapted scale from Rosman, Mayer, Merk, and Kerwer (2019) was employed (see preregistration in Supplementary Materials). The scale consisted of three items relating to the perceived contradictoriness of the administered text materials (α = .82; *r*_it_ between .511 and .756; clear one-dimensional solution in an exploratory factor analysis). High scores indicate stronger perceived contradictoriness—which is why we expected EG3 to score higher on this scale than EG1 (“pro acupuncture” texts) and EG2 (“against acupuncture” texts). This expectation was again supported—descriptively (EG1: *M* = 2.28, *SD* = 1.00; EG2: *M* = 2.48, *SD* = 1.08; EG3: *M* = 3.67, *SD* = 1.07) and in a one-factorial analysis of variance, *F*(2, 898) = 155.75, *p* < .001, $\eta_{p}^{2}$ = .26, all relevant Tukey HSD post-hoc tests *p* < .001.

#### Covariates

Several covariates (e.g., demographics), which were not relevant for our preregistered hypotheses, were additionally measured (see preregistration in Supplementary Materials). These include trust in science (measured at the very beginning of the questionnaire), strength of prior beliefs on acupuncture (measured directly before reading), epistemic emotions while reading (measured directly after the manipulation checks), and beliefs in conspiracy theories (measured at the very end of the questionnaire).

### Operational Hypotheses

Since it is difficult to specify precise hypotheses without explaining a study’s design, the following operational hypotheses were preregistered to complement the general hypotheses suggested in the introduction section:

*Hypothesis 1:* In the quasiexperimental group QEG1 (prior beliefs “pro acupuncture”), SIE will be stronger when participants are confronted with “against acupuncture” texts (EG2) compared to “pro acupuncture” texts (EG1).

We thereby chose to only investigate this hypothesis in the QEG1 group (prior beliefs “pro acupuncture”) since a confrontation with belief-disconfirming evidence is not possible in individuals having no clear opinion on the topic at hand (QEG2 group).

*Hypothesis 2a:* In the quasiexperimental group QEG2 (prior beliefs “no opinion”), there is an interaction between the experimental factor and the CAEB: With increasing scores on the CAEB (both texture [H2a1] and variability^5^5As outlined above, we did not test H2a2 and H2b2 due to the corresponding scale’s insufficient reliability. [H2a2]), SIE will be stronger in EG1 (“pro acupuncture” texts) and EG2 (“against acupuncture” texts) compared to EG3.

*Hypothesis 2b:* The effects specified in H2a1 and H2a2 persist when including all participants and specifying the quasiexperimental factor as a control variable in the respective analyses (H2b1 and H2b2).

In these hypotheses, we focused on participants with no clear opinion on the topic at hand (QEG2 group) to rule out that our results become biased by prior beliefs on the topic of acupuncture.

*Hypothesis 3a:* In the quasiexperimental group QEG2 (prior beliefs “no opinion”), SIE will be stronger in EG3 compared to EG1 (“pro acupuncture” texts; H3a1) and in EG3 compared to EG2 (“against acupuncture” texts; H3a2).

*Hypothesis 3b:* The effects specified in H3a1 and H3a2 persist when including all participants and specifying the quasiexperimental factor as a control variable in the respective analyses (H3b1 and H3b2).

Just as in Hypothesis 2, we focused on participants with no clear opinion on the topic at hand (QEG2 group) to rule out possible confounding effects of prior beliefs on acupuncture.

## Results

Table 3 contains descriptive statistics and intercorrelations of study variables. No gender differences were found on any variable relevant for the present analyses (all *p* > .10). As specified in our preregistration, we used the standard *p* < .05 inference criterion for all analyses, and referred to one-sided tests where appropriate. All analyses were conducted in SPSS (Version 26). A dataset which allows to replicate all the present findings as well as the corresponding syntax and output files can be found in Supplementary Materials.

**Table 3:** Descriptives and Intercorrelations of the Study Variables

Experimental factor	Quasiexperimental factor	Scale	*n*	*M*	*SD*	*r*_SIE-CAEBt_
EG1 (“pro acupuncture” texts)	QEG1 (prior beliefs “pro acupuncture”)	SIE	153	2.68	1.05	.20*
		CAEB_Texture_	153	2.47	0.56	-
	QEG2 (prior beliefs “no opinion”)	SIE	145	2.95	0.92	.46**
		CAEB_Texture_	145	2.84	0.54	-
EG2 (“against acupuncture” texts)	QEG1 (prior beliefs “pro acupuncture”)	SIE	147	3.08	0.99	.10
		CAEB_Texture_	147	2.45	0.63	-
	QEG2 (prior beliefs “no opinion”)	SIE	150	2.90	1.06	.00
		CAEB_Texture_	149	2.92	0.64	-
EG3 (texts “conflicting evidence”)	QEG1 (prior beliefs “pro acupuncture”)	SIE	151	3.57	0.94	.02
		CAEB_Texture_	151	2.40	0.60	-
	QEG2 (prior beliefs “no opinion”)	SIE	155	3.76	0.77	.06
		CAEB_Texture_	155	2.86	0.54	-

*Note.* *n* = sample size per group (after cleaning); *M* = mean; *SD* = standard deviation; SIE = scientific impotence excuse; CAEB_Texture_ = epistemic beliefs on the texture of knowledge; *r*_SIE-CAEBt_ = correlation between CAEB_Texture_ and SIE.

*p < .05. **p < .01.

### Hypothesis 1

Hypothesis 1 was tested using a *t*-test for independent samples in the QEG1 (“pro acupuncture beliefs” group) data subset (dependent variable: SIE; independent variable: experimental factor [EG1, EG2]). The test revealed a significant difference between EG1 (“pro acupuncture” texts) and EG2 (“against acupuncture” texts) regarding SIE, *t*(298) = 3.39, *p* < .001, which was in the expected direction (EG1: *M* = 2.68, *SD* = 1.05; EG2: *M* = 3.08, *SD* = 0.99) and of small to medium effect size (Cohen’s *d* = 0.39; 90% CI [0.20, 0.58]). In other words, participants who read belief-disconfirming information reported higher SIEs compared to participants who read belief-confirming information. Consequently, Hypothesis 1 is fully supported.

### Hypothesis 2

**Figure 1:**
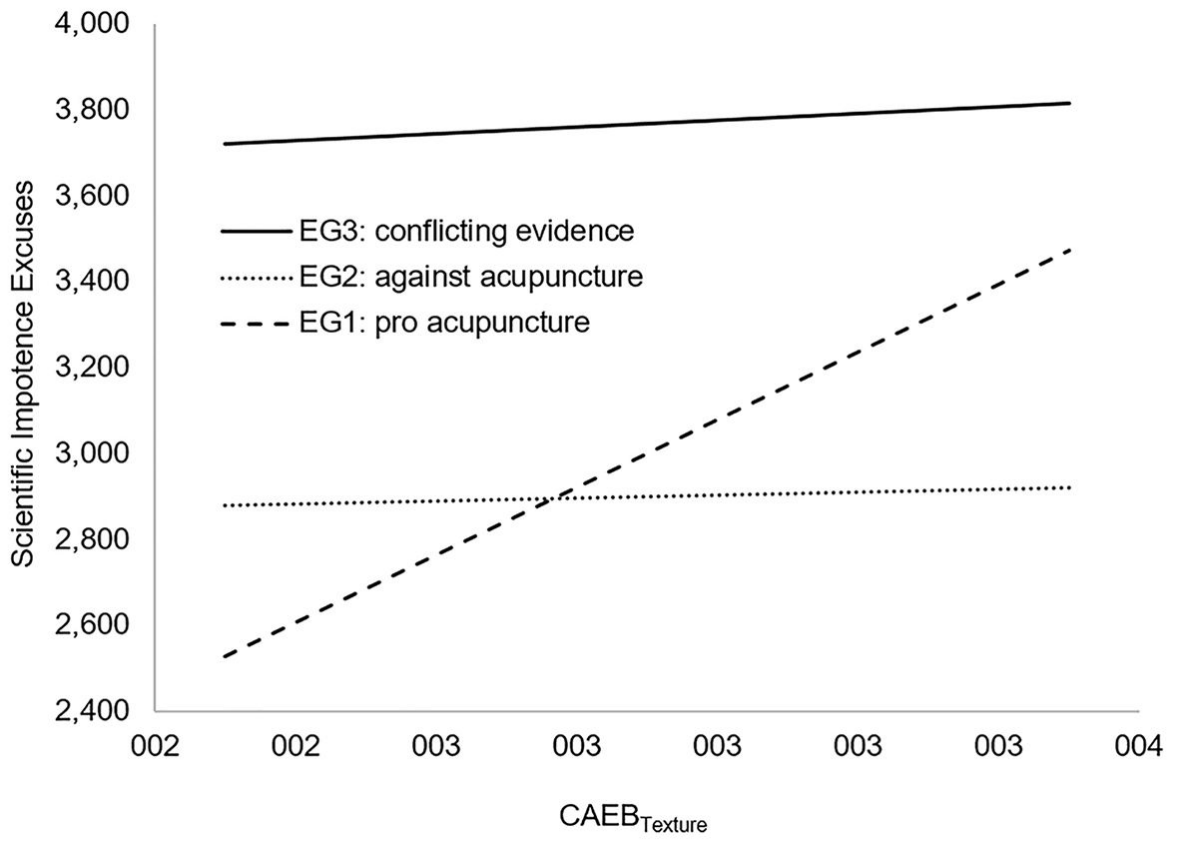
Interaction Between Experimental Conditions (EG1, EG2, EG3) and Epistemic Beliefs (CAEB_Texture_) on Scientific Impotence Excuses for QEG2 (Subjects Who Reported “no opinion” in Their Prior Beliefs on Acupuncture)

Hypothesis 2a1 was tested using regression-based interaction testing (Aiken & West, 1991) by means of the PROCESS 3.4 SPSS macro (Hayes, 2018) in the QEG2 (subjects who reported “no opinion” in their prior beliefs) data subset (PROCESS configuration: Model 1; dependent variable: SIE; independent variable: CAEB_texture_; moderator: experimental condition [EG1, EG2, EG3]). In these analyses, the experimental factor was dummy coded with EG3 (conflicting evidence) as reference category, and CAEB_texture_ was standardized to enhance the interpretability of the results. As can be seen in Table 4, a significant and positive interaction between CAEB_texture_ and the dummy variable for EG1 (“pro acupuncture” texts) was found, which indicates that the slopes of EG1 and EG3 significantly differ (i.e., that effects of epistemic beliefs on SIE differed between individuals who read “pro acupuncture” texts and conflicting evidence), thus supporting H2a1. However, contrary to our expectations, no significant (*p* > .64) interaction was found between CAEB_texture_ and the dummy variable for EG2 (“against acupuncture” texts). Moreover, we only found significant effects of CAEB_texture_ on SIE in the EG1 (“pro acupuncture” texts) group (*B* = 0.45, *p* < .001), but no significant (*p* > .55) effect in EG3 (where we would, according to the consistency hypothesis, have expected a negative effect; see Figure 1 for an illustration of these results). In other words, participants who viewed knowledge as more tentative reported higher SIEs when presented with “pro acupuncture” texts, but not when presented with “against acupuncture” texts. The amount of incremental variance explained by including the interaction between CAEB_texture_ and the experimental condition into the model was Δ*R*^2^ = .039. Finally, we were not able to test H2a2 due to the variability scale’s very low reliability (see above). Hence, while these results provide limited support for H2a1, Hypothesis 2a can only be seen as “partially confirmed.”

**Table 4:** Results of the Multiple Regression Analysis to Test the Interaction between Experimental Condition and Epistemic Beliefs on Scientific Impotence Excuses for Subjects in QEG 2 (No Prior Opinion)

Variable	Scientific impotence excuses			
	Coefficient	*p*	90% CI	
			*LL*	*UL*
CAEB_Texture_	0.04	.56	−0.08	0.17
Against acupuncture (dummy)	−0.86	< .001	−1.03	−0.69
Pro acupuncture (dummy)	−0.79	< .001	−0.96	−0.62
CAEB_Texture_ x Against acupuncture (interaction)	−0.05	.65	−0.21	0.12
CAEB_Texture_ x Pro acupuncture (interaction)	0.41	< .001	0.23	0.59

*Note. N* = 449, *R*^2^ = .22, *F* = 24.40, ∆*R*^2^ when including the interactions = .04. *p*-values are two-tailed. *LL* = lower limit; *UL* = upper limit; CI = confidence interval; CAEB_Texture_ = epistemic beliefs on the texture of knowledge (standardized); dependent variable = scientific impotence excuses.

Hypothesis 2b1 was tested using the same regression-based approach as in H2a. This time, all participants (except those meeting the exclusion criteria) were included in the analysis, and the quasiexperimental factor was included as an additional moderator (PROCESS configuration: Model 2; dependent variable: SIE; independent variable: CAEB_texture_; Moderator 1: experimental condition [EG1, EG2, EG3]; Moderator 2: quasiexperimental factor [QEG1, QEG2]). The experimental factor was, once more, dummy coded with EG3 (conflicting evidence) as reference category and the quasiexperimental factor was dummy-coded with QEG1 (“pro acupuncture beliefs” group) as reference category (hence coded as 0). As in H2a, CAEB_texture_ was standardized before the analysis. The result pattern of this analysis (see Table 5) was practically identical to the results of H2a1: The interaction between CAEB_texture_ and EG1 (“pro acupuncture” texts) was again significant (*B* = 0.30, *p* < .001) whereas no significant interaction between CAEB_texture_ and EG2 (“against acupuncture” texts) was found (*p* > .45). Moreover, no significant interaction between CAEB_texture_ and the quasiexperimental factor was found (*p* > .50). The significant effects of CAEB_texture_ on SIE in the EG1 (“pro acupuncture” texts) group remained significant across the two levels of the quasiexperimental factor (QEG1: *B* = 0.34, *p* < .001; QEG2: *B* = 0.39, *p* < .001), while CAEB_texture_ had no significant effect in the other experimental groups (against acupuncture and conflicting evidence). In other words, increasing views on acupuncture knowledge as tentative seems to lead to a higher amount of SIEs when individuals are presented with “pro acupuncture” texts—regardless of their prior opinion on acupuncture. The amount of incremental variance explained by including the interaction between CAEB_texture_ and the experimental condition was Δ*R*^2^ = .022. H2b2 was not tested because of the above-mentioned reliability issues of the variable scale. The general interpretation of Hypothesis 2b is thus identical to Hypothesis 2a (“partially confirmed,” but only for texture and only for the difference between EG1 and EG3).

**Table 5:** Results of the Multiple Regression Analysis to Test the Interaction Between Experimental Condition, Epistemic Beliefs, and the Quasiexperimental Factor on Scientific Impotence Excuses

Variable	Scientific impotence excuses			
	Coefficient	*p*	90% CI	
			*LL*	*UL*
CAEB_Texture_	0.04	.50	−0.06	0.15
Against acupuncture (dummy)	−0.68	< .001	−0.81	−0.55
Pro acupuncture (dummy)	−0.85	< .001	−0.98	−0.73
QEG2 (dummy)	0.01	.92	−0.10	0.12
CAEB_Texture_ x Against acupuncture (interaction)	−0.06	.45	−0.18	0.07
CAEB_Texture_ x Pro acupuncture (interaction)	0.30	< .001	0.17	0.43
CAEB_Texture_ x QEG (interaction)	0.05	.50	−0.07	0.16

*Note. N* = 900, *R*^2^ = .17, *F* = 25.57, ∆*R*^2^ when including all interactions = .02. Dependent variable = scientific impotence excuses; CAEB_Texture_ = epistemic beliefs on the texture of knowledge (standardized); *LL* = lower limit; *UL* = upper limit; CI = confidence interval.

### Hypothesis 3

Hypothesis 3a was tested using a one-factorial univariate analysis of variance in the QEG2 (“no opinion” group) data subset (dependent variable: SIE; independent variable: experimental factor [EG1, EG2, EG3]). Results revealed highly significant differences in SIE between the experimental groups, *F*(2, 447) = 41.88, *p* < .001, a large effect size ($\eta_{p}^{2}$ = .16, 90% CI [0.11, 0.21]), and all relevant Tukey HSD post-hoc tests were significant (*p* < .001). Descriptively, these differences were in line with our expectations (EG1, “pro acupuncture” texts: *M* = 2.95, *SD* = 0.92; EG2, “against acupuncture” texts: *M* = 2.90, *SD* = 1.06; EG3, Conflicting Evidence: *M* = 3.76, *SD* = 0.77); SIEs were stronger when participants received conflicting evidence compared to consistent evidence pro or against acupuncture (for participants who had no prior opinion on this topic). Hypothesis 3a is therefore fully supported.

**Figure 2:**
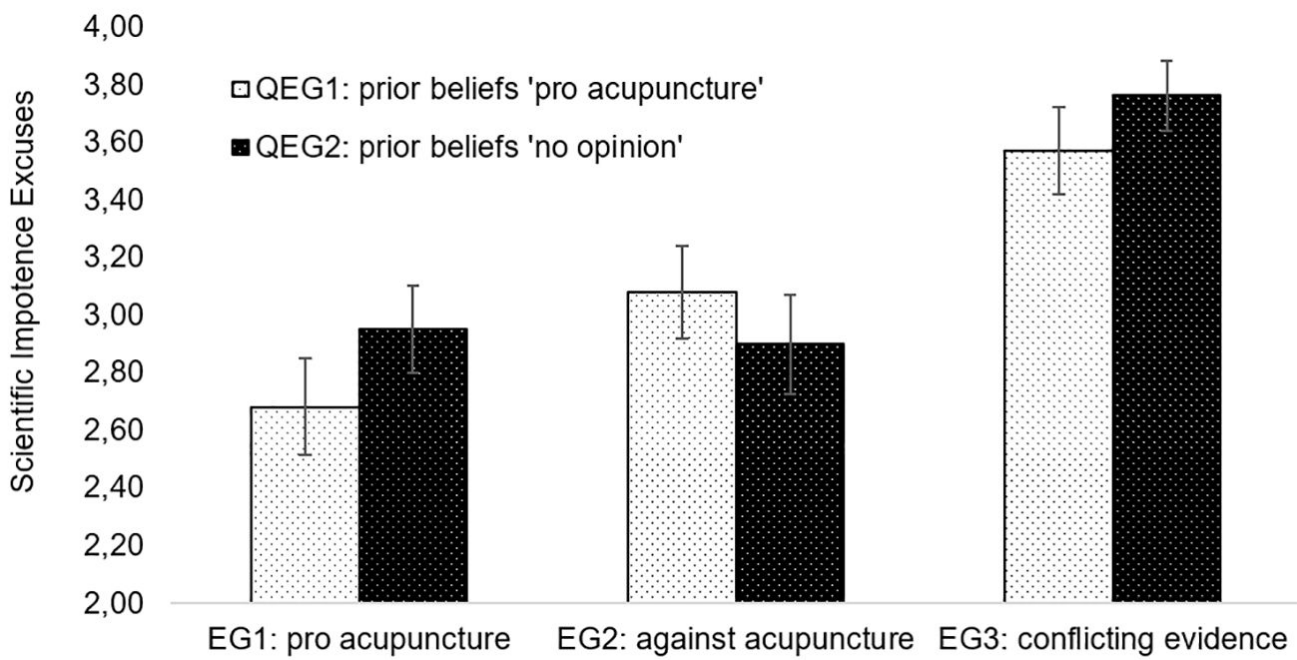
Interaction Between Experimental Conditions (EG1, EG2, EG3) and Quasiexperimental Groups (QEG1, QEG2) on Scientific Impotence Excuses, Including Error Bars With 95% Confidence Intervals

Hypothesis 3b was tested by means of a two-factorial univariate analysis of variance. This time, all participants (except those meeting the exclusion criteria) were included in the analysis, and the quasiexperimental factor was additionally included (dependent variable: SIE; Independent variables: experimental factor [EG1, EG2, EG3], quasiexperimental factor [QEG1, QEG2]). The analysis revealed a result pattern largely similar to H3a, with the main effect of the experimental condition remaining significant, *F*(2, 895) = 66.55, *p* < .001, and the effect size remained large ($\eta_{p}^{2}$ = .13, 90% CI [0.10, 0.16]) when including the interaction between the experimental and the quasiexperimental factor into the model (see Figure 2 for confidence intervals of mean scores in the respective groups). Hypothesis 3b is thus fully supported—SIEs were stronger when participants received conflicting evidence compared to consistent evidence for or against acupuncture regardless of their prior opinion on this topic.

### Exploratory Analyses

An additional look at the results regarding Hypothesis 3 revealed that the interaction between the experimental and the quasiexperimental factor was significant, too, *F*(2, 895) = 4.72, *p* < .01, $\eta_{p}^{2}$ = .01. As can be seen in Figure 2, this interaction seems to be caused by the difference in SIE between EG1 (“pro acupuncture” texts) and EG2 (“against acupuncture” texts) being somewhat stronger in participants believing in acupuncture (QEG1) compared to participants having no clear opinion (QEG2). Participants who had no prior opinion made comparable use of SIEs irrespectively of whether pro or contra acupuncture evidence was presented, whereas participants who believed in acupuncture made more use of SIEs when evidence conflicting with their prior beliefs was presented (but interestingly not for conflicting evidence). While we had not preregistered a corresponding hypothesis, this provides some further support for the “traditional” SIE effect specified in Hypothesis 1. We will come back to this additional exploratory finding in the Discussion section.

## Discussion

It is an old saying that some humans only see what they want to see. This surely does not stop at scientific knowledge, especially in times where established research findings (e.g., on climate change) are questioned by populist political agendas. In the present study, we investigated whether a confrontation with belief-disconfirming scientific evidence leads to beliefs that the topic in question is not amenable to scientific investigation—in short: SIEs. Besides our intention to replicate this “traditional” SIE effect, which was first discovered by Munro (2010), we strived to investigate other factors that might mitigate or amplify SIEs. As a situational factor, we focused on the contradictoriness of the evidence in question, and, as a person-specific factor, we tested the role of epistemic beliefs in SIEs.

### Main Findings

In a first step, we aimed to replicate the “traditional” SIE effect in the context of a health-related topic. For this reason, we initially assessed our participants’ prior beliefs on the effects of acupuncture versus massaging in the treatment of back pain. Subsequently, we presented some of our participants with fictitious evidence on the superiority of acupuncture over massaging, whereas others received evidence speaking *against* acupuncture (i.e., suggesting that massaging was more effective in treating back pain). In our first hypothesis, we had expected that participants believing in the superiority of acupuncture would make stronger SIEs when confronted with belief-disconfirming findings (i.e., evidence against acupuncture) compared to belief-confirming evidence. This expectation was fully supported. In an additional exploratory analysis, we investigated whether this pattern of results differed between participants believing in the superiority of acupuncture and participants having no clear opinion on the topic at hand. The corresponding interaction was significant, revealing some interesting results (see Figure 2): When presented with belief-disconfirming evidence, participants believing in the superiority of acupuncture reported more SIEs compared to participants having no clear opinion, and when presented with belief-confirming evidence, these participants reported even less SIEs than the “no opinion” group. Especially the latter finding is noteworthy—not only do individuals tend to discount science when presented with belief-disconfirming evidence, their beliefs in the potency of science also seem to increase when reading belief-confirming evidence. This is in line with earlier research by Lord et al. (1979), who found that individuals perceived “results and procedures that confirmed their own beliefs to be the more convincing and probative ones” (p. 2098). Thus, additional confirmatory studies are necessary to determine if Munro’s (2010) proposition of an effect which results in (merely) *discounting* science might be a bit short-sighted.

With regard to our second hypothesis, we strived to extend the general SIE proposition to the level of epistemic beliefs—hence we were no longer interested in the effects of content-related beliefs on a certain topic, but in the effects of beliefs regarding the *nature* of knowledge on the respective topic. We expected that SIEs would be stronger when individuals were confronted with scientific evidence exhibiting a “nature” contradicting their (topic-specific) epistemic beliefs, and that these effects would persist when controlling for “traditional” SIE effects. However, our results, especially regarding the former proposition, were somewhat mixed. First, we could not conduct any analyses on the variability subscale of our epistemic beliefs instrument due to reliability issues. Moreover, the hypothesis was only supported with regard to differences between participants presented with conflicting evidence and participants reading evidence favoring acupuncture, whereas no significant differences in the effects of epistemic beliefs were found between participants reading conflicting evidence and participants reading evidence against acupuncture (see Figure 1). This might be caused by the design of our reading task—the information that acupuncture is explicitly better than massaging might be perceived as a stronger message compared to the information that acupuncture is (just) inferior to massaging (but may be effective nonetheless).

Furthermore, an analysis of the slopes in the respective groups revealed a somewhat unexpected result: According to the consistency hypothesis (on which Hypothesis 2 is based; Muis & Franco, 2010; Muis et al., 2011), we would have expected decreasing SIE scores with increasing scores on the CAEB when presenting participants with conflicting evidence. This was, however, clearly not supported by our data (see also Figure 1). Considering these findings, we see the evidence for Hypothesis 2 as somewhat limited. The consistency hypothesis might thus not be as easily adaptable with regard to SIEs as we had initially expected—which is, in hindsight, not so much surprising when taking a closer look at its suspected psychological mechanisms. In fact, to explain the positive effects of consistency on learning, Muis et al. (2011) argue that “during learning, an individual will focus more on aspects of the content that are consonant with that individual’s epistemic profile” (p. 50). Trevors et al. (2017) further elaborate on this by stating that “given a context with an abundance of epistemically consistent information (e.g., more rational information available when solving a math problem), individuals will have more opportunity to reflect on and regulate their unfolding comprehension of this information” (p. 109). While this suspected working mechanism has not yet been tested empirically, we concede that it is hard to argue how an increased or reduced focus on the information at hand would lead to SIEs. Therefore, we might have been overly optimistic regarding the generalizability of the consistency hypothesis when specifying our preregistered hypotheses.

Nonetheless, the finding that stronger epistemic beliefs in the subjectivity of knowledge might hinder individuals in dealing with scientific information is not unprecedented. Prior research showed that this type of beliefs might be negatively related to multiple document comprehension (Bråten et al., 2013) or viewpoint integration (Barzilai & Eshet-Alkalai, 2015). That well-supported knowledge claims (consistent information in favor of acupuncture) in our study resulted in a higher amount of scientific impotency excuses for participants who viewed knowledge on acupuncture as evolving and tentative, should therefore by no means disregarded but might prove to be a fruitful starting point for future research in this area.

In our third hypothesis, we predicted that SIEs would be stronger when individuals are confronted with contradictory scientific evidence compared to non-contradictory evidence. In line with the idea that SIEs relate to the epistemology of a domain, we analyzed this hypothesis by experimentally manipulating the epistemology of the findings presented to our participants—by portraying scientific knowledge on acupuncture versus massaging as inconsistent and conflicting. This hypothesis was fully supported: Individuals presented with conflicting evidence reported much stronger SIEs compared to individuals reading non-conflicting findings. Furthermore, from an exploratory point of view, the descriptive differences in effect sizes between Hypothesis 3 (large effect) and Hypothesis 1 (small to medium effect) are noteworthy. In fact, these differences suggest that the underlying epistemology of the domain in question may more strongly affect SIEs compared to interactions between prior beliefs and the evidence at hand. Related to this, one may question whether SIE-items such as “This topic is not amenable to scientific investigation” (see preregistration in Supplementary Materials) imply SIEs per se, or whether speaking of such an excuse aspect is only justified when there is an explicit connection to belief-disconfirming evidence. This, of course, relates to how broadly or narrowly one conceptualizes the construct in question. Broader conceptualizations are probably more generalizable, whereas narrower conceptualizations are more precise, which is why choosing between the one or the other is challenging. A way out of this dilemma would be a change in terminology from “scientific impotence excuses” to “scientific impotence beliefs,” but for the present article, we decided to stick to the established terminology. Nevertheless, we think that the rather low correlations between epistemic beliefs and our SIE scale (see Table 3) warrant further research on the “broader” construct as they suggest that scientific impotence beliefs are sufficiently different from other epistemology-related beliefs.

### Strengths, Limitations, and Future Directions

A clear strength of our study is its large and heterogeneous sample, which includes participants from a broad range of age and educational settings. Moreover, due to an elaborate quota configuration, we ensured that gender distributions were very similar across the experimental groups, hence making gender bias very unlikely. Nevertheless, it should be mentioned that our study was limited to one single topic from alternative medicine, namely the treatment of back pain via acupuncture versus massaging, and that participants believing in the superiority of massaging over acupuncture were excluded from the study. As outlined above, individuals who refer to alternative medicine (e.g., acupuncture) are more likely to exhibit a less scientific way of thinking (e.g., Furnham, 2007; Saher & Lindeman, 2005). Since we eliminated those participants who believed that conventional therapies (i.e., massaging) are superior to acupuncture, it might thus well be that “unscientific” thinkers were over-represented in our sample. As this may impair the generalizability of our findings, future studies should strive to replicate our findings with regard to different topics and in different samples.

Furthermore, it should be noted that all data were collected using self-reports. As evidenced by our reliability problems regarding the variability scale of the CAEB, there are numerous challenges associated with assessing a complex construct such as epistemic beliefs by this means (DeBacker et al., 2008; Greene & Yu, 2014; Mason, 2016), which is why future research should investigate whether the partially unexpected results regarding Hypothesis 2 may have been caused by measurement issues.

A final limitation constitutes the fact that our analyses do not allow any inferences regarding the psychological mechanisms behind scientific excuses. In other words, our study shows how to trigger SIEs, but does not allow to straighten out which psychological processes are responsible for this triggering. Cognitive dissonance (Festinger, 1957) might be one such mechanism. As outlined above, individuals who experience a conflict between their prior beliefs and the evidence at hand experience feelings of psychological discomfort and strive to reduce these using various strategies—and SIE may be one such strategy (Munro, 2010). Furthermore, directional goals (i.e., the desire to arrive at a particular conclusion) may play a role as it leads to biases in research evaluation (Kunda, 1990). Future research might further investigate this by referring to a process-oriented approach (e.g., mediation analysis), which would require the explicit measurement of, for example, cognitive dissonance.

### Conclusion

Research on SIEs is still very young. Nevertheless, the general SIE proposition has now been conceptually replicated several times, and, importantly, this was done by independent researchers, in varying samples, regarding different topics, and using various designs and measurement instruments. Our study advances this research by replicating the SIE proposition in a large general population sample and by identifying factors that may additionally contribute to SIEs, such as epistemic beliefs or the epistemic nature of the evidence. Considering the accumulating evidence on individuals making SIEs when confronted with belief-disconfirming evidence, it should however also be noted that such excuses are only one example for a devaluation and denigration of science. Therefore, future research should investigate interactions between SIEs and other defense mechanisms (or resistance processes; Munro, 2010), such as simply ignoring belief-disconfirming information (Chinn & Brewer, 1993) or selectively exposing oneself to belief-confirming information (e.g., Hart et al., 2009; Meppelink et al., 2019). Especially the latter two mechanisms may, in times of filter bubbles and echo chambers (Flaxman et al., 2016), be particularly problematic since they are actively supported by technology.

As for practical implications, university students—as future scientists—should be prepared to face SIEs from the public and media, especially when dealing with a rather controversial topic. We therefore recommend teaching them early on how to meet this challenge, and to be clear and reasonable about what kind of knowledge a specific research method can generate and where it reaches its limits. We furthermore think that scientists should make it as hard as possible for the public to come to SIEs—by doing their absolute best in conducting robust, reproducible research, but also by being open and transparent about their work (Munafò et al., 2017; Nosek et al., 2015). Especially the latter aspect is doubtlessly suited to convey a realistic image of science—and we all hope that that is an image of potency.

## Data Availability

Data for this article is freely available (see the Supplementary Materials  section). The full data set (for Study 1) is not available, as national data sharing rules do not presently allow this.

## Supplementary Materials

For this article the following Supplementary Materials are available via PsychArhives (for access see Index of Supplementary Materials below):

- Preregistration documentation.
- A dataset which allows to replicate all the present findings.
- Corresponding syntax and output files.
